# Analytical expression of Kondo temperature in quantum dot embedded in Aharonov-Bohm ring

**DOI:** 10.1186/1556-276X-6-604

**Published:** 2011-11-23

**Authors:** Ryosuke Yoshii, Mikio Eto

**Affiliations:** 1 Faculty of Science and Technology, Keio University, 3-14-1 Hiyoshi, Kohoku-ku, Yokohama 223-8522, Japan

## Abstract

We theoretically study the Kondo effect in a quantum dot embedded in an Aharonov-Bohm ring, using the "poor man's" scaling method. Analytical expressions of the Kondo temperature *T*_K _are given as a function of magnetic flux Φ penetrating the ring. In this Kondo problem, there are two characteristic lengths, $L_{\text{c}}=\hbar v_{\text{F}}∕|{\tilde{\varepsilon }}_{0}|$ and *L*_K _= *ħv*_F _= *T*_K_, where *v*_F _is the Fermi velocity and ${\tilde{\varepsilon }}_{0}$ is the renormalized energy level in the quantum dot. The former is the screening length of the charge fluctuation and the latter is that of the spin fluctuation, i.e., size of Kondo screening cloud. We obtain diferent expressions of *T*_K_(Φ) for (i) *L*_c _≪ *L*_K _≪ *L*, (ii) *L*_c _≪ *L *≪ *L*_K_, and (iii) *L *≪ *L*_c _≪ *L*_K_, where *L *is the size of the ring. *T*_K _is remarkably modulated by Φ in cases (ii) and (iii), whereas it hardly depends on Φ in case (i).

PACS numbers:

## Introduction

Since the first observation of the Kondo effect in semiconductor quantum dots [1-3], various aspects of Kondo physics have been revealed, owing to the artificial tunability and flexibility of the systems, e.g., an enhanced Kondo effect with an even number of electrons at the spin-singlet-triplet degeneracy [4], the SU(4) Kondo effect with *S *= 1/2 and orbital degeneracy [5], and the bonding and antibonding states between the Kondo resonant levels in coupled quantum dots [6,7]. One of the major issues which still remain unsolved in the Kondo physics is the observation of the Kondo singlet state, so-called Kondo screening cloud. The size of the screening cloud is evaluated as *L*_K _= *ħv*_F_/*T*_K_, where *v*_F _is the Fermi velocity and *T*_K _is the Kondo temperature. There have been several theoretical works on *L*_K_, e.g., ring-size dependence of the persistent current in an isolated ring with an embedded quantum dot [8], Friedel oscillation around a magnetic impurity in metal [9], and spin-spin correlation function [10,11].

We focus on the Kondo effect in a quantum dot embedded in an Aharonov-Bohm (AB) ring. In this system, the conductance shows an asymmetric resonance as a function of energy level in the quantum dot, so-called Fano-Kondo effect. This is due to the coexistence of one-body interference effect and many-body Kondo effect, which was studied by the equation-of-motion method with the Green function [12], the numerical renormalization group method [13], the Bethe ansatz [14], the density-matrix renormalization group method [15], etc. This Fano-Kondo resonance was observed experimentally [16]. The interference effect on the value of *T*_K_, however, has not been fully understood [17,18].

In our previous work [19], we examined this problem in the small limit of AB ring using the scaling method [20]. Our theoretical method is as follows. First, we create an equivalent model in which a quantum dot is coupled to a single lead. The AB interference effect is involved in the flux-dependent density of states in the lead. Second, the two-stage scaling method is applied to the reduced model, to renormalize the energy level in the quantum dot by taking into account the charge fluctuation and evaluate *T*_K _by taking spin fluctuation [21]. This method yields *T*_K _in an analytical form.

The purpose of this article is to derive an analytical expression of *T*_K _for the finite size of the AB ring, using our theoretical method. We find two characteristic lengths. One is the screening length of the charge fluctuation, $L_{\text{c}}=\hbar v_{\text{F}}∕|{\tilde{\varepsilon }}_{0}|$ with ${\tilde{\varepsilon }}_{0}$ being the renormalized energy level in the quantum dot, which appears in the first stage of the scaling. The other is the size of Kondo screening cloud, *L*_K_, which is naturally obtained in the second stage. In consequence, the analytical expression of *T*_K _is different for situations (i) *L*_c _≪ *L*_K _≪ *L*, (ii) *L*_c _≪ *L *≪ *L*_K_, and (iii) *L *≪ *L*_c _≪ *L*_K_, where *L *is the size of the ring. We show that *T*_K _strongly depends on the magnetic flux Φ penetrating the AB ring in cases (ii) and (iii), whereas it hardly depends on Φ in case (i).

## Model

**Figure 1:**
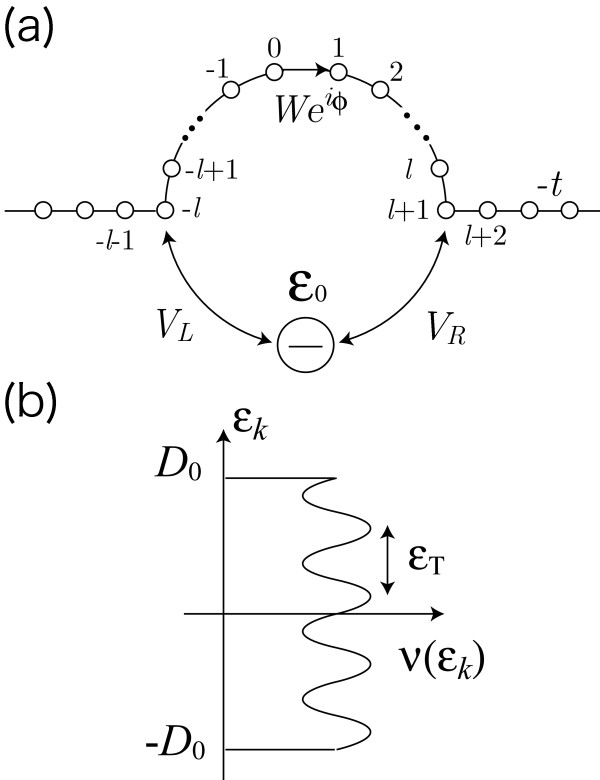
**(a) Model for an Aharonov-Bohm (AB) ring with an embedded quantum dot**. A quantum dot with an energy level *ε*_0 _is connected to two external leads by tunnel couplings, *V_L _*and *V_R_*. Another arm of the AB ring (reference arm) and external leads are represented by a one-dimensional tight-binding model. The reference arm includes a tunnel barrier with transfer integral *W*. The magnetic flux Φ penetrating the ring is considered as an AB phase *ϕ *= 2*π*Φ/Φ_0 _with flux quantum Φ_0 _= *h*/*e*. **(b) **The density of states in the lead for the reduced model, *ν*(*ε_k_*) in Eq. (6). *ν*(*ε_k_*) oscillates with the period of *ε*_T_, the Thouless energy for the ballistic systems. Its amplitude and phase depend on the AB phase *ϕ*.

Our model is shown in Figure 1a. A quantum dot with an energy level *ε*_0 _is connected to two external leads by tunnel couplings, *V*_L _and *V*_R_. Another arm of the AB ring (reference arm) and external leads are represented by a one-dimensional tight-binding model with transfer integral -*t *and lattice constant *a*. The size of the ring is given by *L *= (2*l *+ 1)*a*. The reference arm includes a tunnel barrier with transmission probability of *T*_b _= 4*x*/(1 + *x*)^2 ^with *x *= (*W*/*t*)^2^. The AB phase is denoted by *ϕ *= 2*π*Φ/Φ_0_, with flux quantum Φ_0 _= *h*/*e*. The Hamiltonian is

(1) $$H^{\left(0\right)}=H_{\text{dot}}+H_{\text{leads + ring}}+H_{\text{T}},$$

(2) $$H_{\text{dot}}=\underset{\sigma }{\sum }\varepsilon_{0}d_{\sigma }^{†}d_{\sigma }+U{\hat{n}}_{↑}{\hat{n}}_{↓},$$

(3) $$\begin{array}{llll} H_{\text{leads + ring}}= & \underset{i\neq 0}{\sum }\underset{\sigma }{\sum }\left(-ta_{i+1,\sigma }^{†}a_{i,\sigma }+\text{h}.\text{c}.\right)\; & & \;\\ & +\underset{\sigma }{\sum }\left(We^{i\phi }a_{1,\sigma }^{†}a_{0,\sigma }+\text{h}.\text{c}.\right),\; & & \;\\ & \; & \end{array}$$

(4) $$H_{\text{T}}=\underset{\sigma }{\sum }\left(V_{\text{L}}d_{\sigma }^{†}a_{-l,\sigma }+V_{\text{R}}d_{\sigma }^{†}a_{l+1,\sigma }+\text{h}.\text{c}.\right),$$

where $d_{\sigma }^{†}$ and *d_σ _*are creation and annihilation operators, respectively, of an electron in the quantum dot with spin σ. $a_{i,\sigma }^{†}$ and *a*_*i*,*σ *_are those at site *i *with spin *σ *in the leads and the reference arm of the ring. ${\hat{n}}_{\sigma }=d_{\sigma }^{†}d_{\sigma }$ is the number operator in the dot with spin *σ*. *U *is the charging energy in the dot.

We consider the Coulomb blockade regime with one electron in the dot, -*ε*_0_, *ε*_0 _+ *U *≫ Γ, where Γ = Γ_L _+ Γ is the level broadening. $\Gamma_{\alpha }=\pi \nu_{0}V_{\alpha }^{2}$, with *ν*_0 _being the local density of states at the end of semi-infinite leads. We analyze the vicinity of the electron-hole symmetry of -*ε*_0 _≈ *ε*_0 _+ *U*.

We create an equivalent model to the Hamiltonian (1), following Ref. [19]. First, we diagonalize the Hamiltonian *H*_leads+ring _for the outer region of the quantum dot. There are two eigenstates for a given wavenumber *k*; |*ψk*,→〉 represents an incident wave from the left and partly reflected to the left and partly transmitted to the right, whereas |*ψ_k_*,←〉 represents an incident wave from the right and partly reflected to the right and partly transmitted to the left. Next, we perform a unitary transformation for these eigenstates

$$(|{\overset{{}^{\circ}}{\psi }}_{k}\rangle \;|\psi_{k}\rangle )=(|\psi_{k,\to }\rangle \;|\psi_{k,\leftarrow }\rangle )\;\left(\begin{array}{cc} A_{k} & B_{k}^{*}\\ B_{k} & -A_{k}^{*} \end{array}\right),$$

where *A_k _*and *B_k _*are determined so that $\langle d|H_{\text{T}}|{\bar{\psi }}_{k}\rangle =0$ with dot state |*d*〉. In consequence, mode |*ψ_k_*〉 is coupled to the dot via *H*_T_, whereas $|{\overset{{}^{\circ}}{\psi }}_{k}\rangle$ is completely decoupled.

Neglecting the decoupled mode, we obtain the equivalent model in which a quantum dot is coupled to a single lead. In a wide-band limit, the Hamiltonian is written as

(5) $$\begin{array}{llll} H= & \underset{\sigma }{\sum }\varepsilon_{0}d_{\sigma }^{†}d_{\sigma }+U{\hat{n}}_{↑}{\hat{n}}_{↓}+\underset{k,\sigma }{\sum }\varepsilon_{k}a_{k,\sigma }^{†}a_{k,\sigma }\; & & \;\\ & +\underset{k,\sigma }{\sum }V\left(d_{\sigma }^{†}a_{k,\sigma }+\text{h}.\text{c}.\right),\; & & \;\\ & \; & \end{array}$$

with $V=\sqrt{V_{\text{L}}^{2}+V_{\text{R}}^{2}}$ and density of states in the lead

(6) $$\nu \left(\varepsilon_{k}\right)=\nu_{0}\left[1-R_{\text{b}}\cos\frac{\varepsilon_{k}+D_{0}}{\varepsilon_{\text{T}}}-P\left(\phi \right)\sin\frac{\varepsilon_{k}+D_{0}}{\varepsilon_{\text{T}}}\right].$$

Here, *D*_0 _is the half of the band width, *k*_F _is the Fermi wavenumber, *R*_b _= 1 - *T*_b_, and

(7) $$P\left(\phi \right)=\sqrt{\alpha T_{\text{b}}\left(1-T_{\text{b}}\right)}\cos\phi ,$$

where *α *= 4Γ_L_Γ_R_/(Γ_L _+ Γ_R_)^2 ^is the asymmetric factor for the tunnel couplings of quantum dot.

The AB interference effect is involved in the flux-dependent density of states in the lead, *υ*(*ε_k_*) in Eq. (6). As schematically shown in Figure 1(b), *υ*(*ε_k_*) oscillates with the period of *ε*_T_, where *ε*_T _= *ħv*_F_/*L *is the Thouless energy for the ballistic systems. We assume that *ε*_T _≪ *D*_0_.

## Scaling analysis

We apply the two-stage scaling method to the reduced model. In the first stage, we consider the charge fluctuation at energies of *D *≫ |*ε*_0_|. In this region, the number of electrons in the quantum dot is 0, 1, or 2. We reduce the energy scale from bandwidth *D*_0 _to *D*_1 _where the charge fluctuation is quenched. By integrating out the excitations in the energy range of *D*_1 _*< D < D*_0_, we renormalize the energy level in the quantum dot *ε*_0_. In the second stage of scaling, we consider the spin fluctuation at low energies of *D < D*_1_. We make the Kondo Hamiltonian and evaluate the Kondo temperature.

## Renormalization of energy level

In the first stage, the charge fluctuation is taken into account. We denote *E*_0_, *E*_1_, and *E*_2 _for the energies of the empty state, singly occupied state, and doubly occupied state in the quantum dot, respectively. Then the energy levels in the quantum dot are given by *ε*_0 _= *E*_1 _- *E*_0 _for the first electron and *ε*_1 _= *E*_2 _- *E*_1 _for the second electron. When the bandwidth is reduced from *D *to *D *- |d*D*|, *E*_0_, *E*_1_, and *E*_2 _are renormalized to *E*_0 _+ d*E*_0_, *E*_1 _+ d*E*_1_, and *E*_2 _+ d*E*_2_, where

$$\begin{array}{c} \text{d}E_{0}=-\frac{2V^{2}\nu \left(-D\right)}{D+E_{1}-E_{0}}|\text{d}D|,\\ \text{d}E_{1}=-\left[\frac{V^{2}\nu \left(D\right)}{D+E_{0}-E_{1}}+\frac{V^{2}\nu \left(-D\right)}{D+E_{2}-E_{1}}\right]|\text{d}D|,\\ \text{d}E_{2}=-\frac{2V^{2}\nu \left(D\right)}{D+E_{1}-E_{2}}|\text{d}D|, \end{array}$$

within the second-order perturbation with respect to tunnel coupling *V*. For *D *≫ |*E*_1 _- *E*_0_|, |*E*_2 _- *E*_1_|, they yield the scaling equations for the energy levels

(8) $$\frac{\text{d}\varepsilon_{i}}{d\ln D}=2\nu_{0}V^{2}f\left(k_{\text{F}}L,\phi \right)\sin\frac{D}{\varepsilon_{\text{T}}},$$

where *i *= 0, 1 and

(9) $$f\left(k_{\text{F}}L,\phi \right)=R_{\text{b}}\sin k_{\text{F}}L-P\left(\phi \right)\cos k_{\text{F}}L.$$

By the integration of the scaling equation from *D*_0 _to $D_{1}≃|{\tilde{\varepsilon }}_{0}|$, we renormalize the energy levels in the quantum dot *ε_i _*to ${\tilde{\varepsilon }}_{1}$:

(10) $${\tilde{\varepsilon }}_{i}-\varepsilon_{i}≃2\nu_{0}V^{2}f\left(k_{\text{F}}L,\phi \right)\left[\text{Si}\left(\frac{\left|\varepsilon_{0}\right|}{\varepsilon_{\text{T}}}\right)-\text{Si}\left(\frac{D_{0}}{\varepsilon_{\text{T}}}\right)\right],$$

where

$$\text{Si}\left(x\right)=\overset{x}{\underset{0}{\int }}\frac{\sin\xi }{\xi }\text{d}\xi .$$

Si(*x*) goes to 0 as *x *→ 0 and *π*/2 as *x *→ ∞.

From Equation 10, we conclude that

(11) $${\tilde{\varepsilon }}_{i}≃\varepsilon_{i}-\pi \nu_{0}V^{2}f\left(k_{\text{F}}L,\phi \right),$$

when $\varepsilon_{\text{T}}\gg |{\tilde{\varepsilon }}_{0}|$, and ${\tilde{\varepsilon }}_{i}=\varepsilon_{i}$ when $\varepsilon_{\text{T}}\ll |{\tilde{\varepsilon }}_{0}|$. These results can be rewritten in terms of length scale. We introduce $L_{\text{c}}=\hbar v_{\text{F}}∕|{\tilde{\varepsilon }}_{0}|$, which corresponds to the screening length of charge fluctuation. When *L *≪ *L*_c_, the renormalized level ${\tilde{\varepsilon }}_{i}$ is given by Equation 11. When *L *≫ *L*_c_, the energy level is hardly renormalized and is independent of *ϕ*.

## Renormalization of exchange coupling

In the second stage, we consider the spin fluctuation at low energies of *D < D*_1_. For the purpose, we make the Kondo Hamiltonian via the Schrieffer-Wolff transformation,

(12) $$H_{\text{Kondo}}=\underset{k,\sigma }{\sum }\varepsilon_{k\sigma }a_{k\sigma }^{†}a_{k\sigma }+H_{J}+H_{K},$$

(13) $$\begin{array}{c} H_{J}=J\sum_{k^{\prime },k}[S^{+}a_{k^{\prime }↓}^{†}a_{k↑}+S^{-}a_{k^{\prime }↑}^{†}a_{k↓}^{†}\\ \;+S_{z}(a_{k^{\prime }↑}^{†}a_{k↑}-a_{k^{\prime }↓}^{†}a_{k↓})], \end{array}$$

(14) $$H_{k}=K\underset{k^{\prime },k}{\sum }\underset{\sigma }{\sum }\sigma_{k^{\prime }\sigma }^{†}a_{k\sigma },$$

where $S^{+}=d_{↑}^{†}d_{↓}$, $S^{-}=d_{↓}^{†}d_{↑}$ and $S_{z}=\left(d_{↑}^{†}d_{↑}-d_{↓}^{†}d_{↓}\right)∕2$ are the spin operators in the quantum dot. The density of states in the lead is given by Equation 6 and half of the band width is now $D_{1}≃|{\tilde{\varepsilon }}_{0}|$. *H_J _*represents the exchange coupling between spin 1/2 in the dot and spin of conduction electrons, whereas *H_K _*represents the potential scattering of the conduction electrons by the quantum dot. The coupling constants are given by

$$J=V^{2}\left(\frac{1}{\left|{\tilde{\varepsilon }}_{0}\right|}+\frac{1}{{\tilde{\varepsilon }}_{1}}\right),\;K=\frac{V^{2}}{2}\left(\frac{1}{\left|{\tilde{\varepsilon }}_{0}\right|}-\frac{1}{{\tilde{\varepsilon }}_{1}}\right).$$

By changing the bandwidth, we renormalize the coupling constants *J *and *K *so as not to change the low-energy physics within the second-order perturbation with respect to *H_J _*and *H_K_*. Then we obtain the scaling equations of

(15) $$\begin{array}{llll} \frac{dJ}{d\ln D}= & -2\nu_{0}J^{2}\left[1-f\left(k_{\text{F}}L+\pi /)2,\varphi \right)cos\frac{D}{\varepsilon_{\text{T}}}\right]\; & & \;\\ & -4\nu_{0}JKf\left(k_{\text{F}}L,\varphi \right)sin\frac{D}{\varepsilon_{\text{T}}},\; & & \;\\ & \; & \end{array}$$

(16) $$\frac{dK}{d\ln D}=-2\nu_{0}\left(\frac{3}{4}J^{2}+4K^{2}\right)f\left(k_{\text{F}}L,\varphi \right)sin\frac{D}{\varepsilon_{\text{T}}},$$

The energy scale *D *where the fixed point (*J *→ ∞) is reached yields the Kondo temperature.

Scaling equations (15) and (16) are analyzed in two extreme cases. In the case of *D *≫ *ε*_T_, the oscillating part of the density of states *ν*(*ε_k_*) is averaged out in the integration [22]. Then the scaling equations are effectively rewritten as

(17) $$\frac{dJ}{d\ln D}≃-2\nu_{0}J^{2},$$

(18) $$\frac{dK}{d\ln D}≃0.$$

In the case of *D *≪ *ε*_T_, the expansion around the fixed point [23] yields

(19) $$\frac{K}{J}≃\frac{3}{8}c,$$

(20) $$2\nu \left(D\right)J=\frac{\left[1+O\left(c^{2}\right)\right]}{\ln\left(1+\xi \right)},$$

where *ξ *= *D*/*T*_K _- 1 and

(21) $$c≃\frac{2f\left(k_{\text{F}}L,\phi \right)}{1-f\left(k_{\text{F}}L,\phi \right)}\frac{D}{\varepsilon_{\text{T}}}.$$

Now we evaluate the Kondo temperature in situations (i) *L*_c _≪ *L*_K _≪ *L*, (ii) *L*_c _≪ *L *≪ *L*_K_, and (iii) *L *≪ *L*_c _≪ *L*_K_, where *L*_K _= *ν*_F_*ħ*/*T*_K_. In situation (i), *ε*_T _≪ *T*_K _and thus *J *and *K *follow Equations 17 and 18 until the scaling ends at *D *≃ *T*_K_. Integration of Equation 17 from *D*_1 _to *T*_K _yields

(22) $$T_{\text{K}}≃|\varepsilon_{0}|\exp\left(-1/)2\nu_{0}J\right)\equiv T_{\text{K}}^{\left(0\right)},$$

where $J=V^{2}\left(|\varepsilon_{0}{|}^{-1}+\varepsilon_{1}^{-1}\right)$.

In situation (iii), *D*_1 _≪ *ε*_T_. Then the scaling equations (19) and (20) are valid in the whole scaling region (*T*_K _<*D *<*D*_1_). From the equations, we obtain

(23) $$T_{\text{K}}\left(\phi \right)≃|\varepsilon_{0}|{\left(T_{\text{K}}^{\left(0\right)}/)|\varepsilon_{0}|\right)}^{f\left(\phi \right)},$$

where *f*(*ϕ*) = [1 - *f*(*k*_F_*L *+ *π*/2, *ϕ*)]^-1^.

In situation (ii), *T*_K _≪ *ε*_T _≪ *D*_1_. The coupling constants, *J *and *K*, are renormalized following Equations 17 and 18 when *D *is reduced from *D*_1 _to *ε*_T _and following Equations 19 and 20 when *D *is reduced from *ε*_T _to *T*_K_. We match the solutions of the respective equations around *D *= *ε*_T _and obtain

(24) $$T_{\text{K}}\left(\phi \right)≃\varepsilon_{\text{T}}e^{\gamma }{\left(T_{\text{K}}^{\left(0\right)}/)\varepsilon_{\text{T}}e^{\gamma }\right)}^{f\left(\phi \right)},$$

where *γ *≈ 0.57721 is the Euler constant.

**Figure 2:**
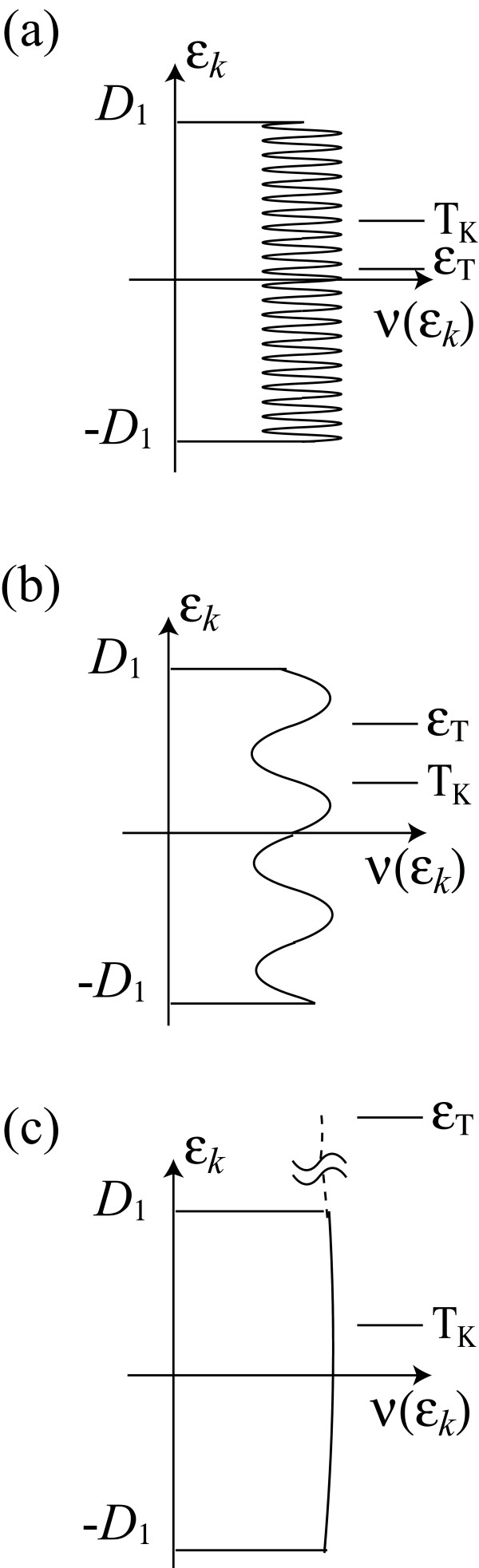
**Schematic drawing of the density of states in the lead for the reduced model, in situations (a) *L*_c _≪ *L*_K _≪ *L*, (b) *L*_c _≪ *L *≪ *L*_K_, and (c) *L *≪ *L*_c _≪ *L*_K_, where *L *is the size of the AB ring, *L*_c _is the screening length of charge fluctuation, and *L*_K _is that of spin fluctuation, i.e., size of Kondo screening cloud**. The half of band width is *D*_1 _≃ |*ε*_0_| in the second stage of scaling. In situation (a), *ε*_T _≪ *T*_K _≪ |*ε*_0_|. The oscillating part of *ν*(*ε_k_*) is averaged out in the integration of scaling equations. In consequence, the Kondo temperature *T*_K _does not depend on the ring size nor AB phase *ϕ *of the magnetic flux penetrating the ring. In situation (b), *T*_K _≪ *ε*_T _≪ |*ε*_0_|. Then the Thouless energy *ε*_T _acts as the high energy cut off. *ϕ*-dependence of *T*_K _is determined by the ratio of *ε*_T _to *T*_K_. In situation (c), *T*_K _≪ |*ε*_0_| ≪ *ε*_T_. The density of states is almost constant. In this case, *T*_K _reflects the density of states at the Fermi level, *ν*(0).

The different expressions of *T*_K_(*ϕ*) in the three situations can be explained intuitively. In situation (i), *ε*_T _≪ *T*_K_. Then the oscillating part of the density of states *ν*(*ε_k_*) with period *ε*_T _is averaged out in the scaling procedure (Figure 2a). As a result, the magnetic-flux dependence of *T*_K _disappears. In situation (iii), *T*_K _≪ *ε*_T_. Then *ν*(*ε_k_*) is almost constant in the scaling (Figure 2c). The Kondo temperature significantly depends on the magnetic flux through the constant value of *ν*(0) at the Fermi level.

## Conclusions

We have theoretically studied the Kondo effect in a quantum dot embedded in an AB ring. The two-stage scaling method yields an analytical expression of the Kondo temperature *T*_K _as a function of AB phase *ϕ *of the magnetic flux penetrating the ring. We have obtained different expressions of *T*_K_(*ϕ*) for (i) *L*_c _≪ *L*_K _≪ *L*, (ii) *L*_c _≪ *L *≪ *L*_K_, and (iii) *L *≪ *L*_c _≪ *L*_K_, where *L *is the size of the ring, $L_{\text{c}}=\hbar v_{\text{F}}∕|{\tilde{\varepsilon }}_{0}|$ is the screening length of the charge fluctuation, and *L*_K _= *ħν*_F_/*T*_K _is the screening length of the charge fluctuation, i.e., size of Kondo screening cloud. *T*_K _strongly depends on *ϕ *in cases (ii) and (iii), whereas it hardly depends on *ϕ *in case (i).

## Abbreviation

AB: Aharonov-Bohm.

## Competing interests

The authors declare that they have no competing interests.

## Authors' contributions

All authors read and approved the final manuscript.
